# Racial Disparities in Necrotizing Enterocolitis

**DOI:** 10.3389/fped.2021.633088

**Published:** 2021-02-18

**Authors:** Alain Cuna, Venkatesh Sampath, Minesh Khashu

**Affiliations:** ^1^School of Medicine, University of Missouri Kansas City, Kansas City, MO, United States; ^2^Division of Neonatology, Children's Mercy Kansas City, Kansas City, MO, United States; ^3^Neonatal Service, University Hospitals Dorset, Poole, United Kingdom; ^4^Bournemouth University, Dorset, United Kingdom

**Keywords:** newborn, prematurity, race, genetics, necrotizing enterocolitis

## Abstract

Necrotizing enterocolitis (NEC) is a serious disease of the intestinal tract affecting 5–10% of pre-term infants with up to 50% mortality in those that require surgery. There is wide variation in the rates and outcomes of NEC by race and ethnicity, and the reasons for this disparity are poorly understood. In this article, we review the epidemiology and discuss possible explanations for racial and ethnic differences in NEC. Most of the current evidence investigating the role of race in NEC comes from North America and suggests that Hispanic ethnicity and non-Hispanic Black race are associated with higher risk of NEC compared to non-Hispanic White populations. Differences in pre-term births, breastfeeding rates, and various sociodemographic factors does not fully account for the observed disparities in NEC incidence and outcomes. While genetic studies are beginning to identify candidate genes that may increase or decrease risk for NEC among racial populations, current data remain limited by small sample sizes and lack of validation. Complex interactions between social and biological determinants likely underly the differences in NEC outcomes among racial groups. Larger datasets with detailed social, phenotypic, and genotypic information, coupled with advanced bioinformatics techniques are needed to comprehensively understand racial disparities in NEC.

## Introduction

Necrotizing enterocolitis (NEC) is a devastating inflammatory disease of the intestinal tract that affects 5–10% of pre-term infants born <1,500 grams at birth. It is the most common cause of emergency gastrointestinal surgery in the neonatal period and is the leading cause of death in pre-term infants between 2 weeks and 2 months of age (1, 2). Several risk factors have been implicated in NEC, including prematurity, small for gestational age, formula feeding, infection, ischemia, host genetics, and gut dysbiosis (3–5). Race has also been identified as an important risk factor for NEC. In the clinical risk index tool called GutCheck^NEC^, non-Hispanic Black or Hispanic race is one of nine independent risk factors that collectively can help identify infants at risk for developing NEC (6).

While recent studies suggest that rates of NEC are decreasing, the racial gap in rates and outcomes of NEC has remained unchanged (7, 8). The reasons for underlying racial differences in pre-term NEC outcomes are poorly understood. Racial disparities in NEC persist despite controlling for several sociodemographic factors, suggesting that more complex factors that drive racial differences in NEC are involved. A more comprehensive understanding of how these factors interact with each other is needed to craft potential solutions that reduce racial disparities in NEC.

In this article, we review the epidemiologic evidence behind racial differences in NEC, discuss possible explanations for these differences, and examine key questions for future research. This article will focus on the disparities in NEC outcomes among non-Hispanic Black, non-Hispanic White, and Hispanic infants in the United States, as these populations are the best studied in the literature.

## Epidemiology of Racial Disparities in NEC

### Racial Disparities in NEC Across the Globe

Epidemiological studies of NEC from different countries provide clues regarding the contribution of race in NEC pathogenesis. In general, NEC appears to occur less frequently among pre-term infants from Japan, Switzerland, and Austria while afflicting pre-term infants more often in North America, the United Kingdom, and Ireland (9). A recent study by Su et al. (10) that compared outcomes of extremely pre-term infants from Taiwan with those from other countries found that NEC rates were noticeably lower in Taiwan (3%) and Japan (3%) compared to Canada (8%) and the United States (11%). While race may explain these differences, other reasons such as variations in neonatal care practices can also explain the marked variation in NEC rates across these different countries. For example, countries with a more active approach for offering intensive care to babies at the limits of viability may have higher NEC rates because of the higher survival of extreme pre-term babies most at risk for NEC, while countries with high usage rates of breastmilk or probiotics may have lower NEC rates (11). Inconsistencies in the criteria used to define NEC may also make NEC rates vary among different countries. However, in a systematic review of NEC incidence from high income countries, Battersby et al. (11) demonstrated an almost 4-fold difference in NEC rates even among countries that used Bell staging ≥2 to define NEC.

### Racial Disparities in NEC in the United States

Additional evidence for the role of race in NEC comes from studies from the United States, where differences in incidence and severity of NEC based on race were observed in both single-center and regional studies where care practices and case definitions for NEC are similar (Table 1).

**Table 1:** Summary of studies showing racial differences in NEC-related outcomes in the United States.

**Study**	**Cohort characteristics**	**Main findings**	**Summary statistics**	**Adjustments to analysis**
Uauy et al. (12)	2,681 very low-birth-weight infants from eight participating University centers of the National Institute of Child Health and Human Development	Increased odds of proven NEC among non-Hispanic Black compared to other male infants	aRR 2.3 (1.5–3.4)	Center
Holman et al. (13)	6,629 infants who died of NEC based on data from National Center for Health Statistics, Centers for Disease Control and Prevention	Increased NEC-related mortality among non-Hispanic Black infants	aRR 1.5 (1.3–1.8)	Birth weight, sex, maternal age
Llanos et al. (14)	85 infants with NEC from two regional perinatal centers in Upstate New York	Increased risk for NEC among non-Hispanic Black compared to non-Hispanic White infants	aRR 1.70 (1.06–2.61)	Birth weight
Holman et al. (15)	4,463 hospitalizations associated with NEC were studied using Kids' Inpatient Database	NEC-related hospitalization rate (per 100,000 live births) higher for non-Hispanic Blacks than for both non-Hispanic Whites and Hispanics	Non-Hispanic Black: 165.6 (137.2–194) Non-Hispanic White: 63.2 (51.5–74.9)	Not applicable
Guner et al. (16)	2,318 infants with NEC from California Office of Statewide Health Planning and Development database	Increased mortality among Hispanic infants with NEC compared to other ethnicities	aOR 1.50 (1.09–2.07)	Birth weight, level of hospital, insurance status, and median household income
Anderson et al. (17)	245,242 pre-term infants from California Office of Statewide Health Planning and Development database	Hispanic infants born at 26–28 weeks' gestation more likely to develop NEC than non-Hispanic White infants	aOR 1.32 (1.05–1.66)	Gestational age, birth weight, sex, multiple gestation
Jammeh et al. (18)	126,089 infants from Pediatrix Medical Group database	Increased NEC incidence and NEC mortality among non-Hispanic Blacks and Hispanic infants compared to non-Hispanic White infants	*NEC incidence* Non-Hispanic Black: aOR 1.31 (1.25–1.39) Hispanic: aOR 1.30 (1.21–1.39) *NEC mortality* Non-Hispanic Black: aOR 1.35 (1.15–1.58) Hispanic: aOR 1.31 (1.09–1.56)	Birth weight, gestational age, gender, size for gestational age, type of enteral feeding, prenatal steroid exposure, ventilator support, and inotropic support
Janevic et al. (19)	582,297 infants from New York State Department of Health and Human Services	Increased risk for NEC in non-Hispanic Black compared to non-Hispanic White infants	Conventional approach: aRR 1.52 (1.10–2.11) Fetus-at-risk approach: aHR 4.40 (2.98–6.51)	Maternal age, parity, educational level, insurance status, sex, maternal morbidities
Goldstein et al. (7)	47,112 infants from California Perinatal Quality Care Collaborative	Increased odds of NEC among Hispanic and Asian/Pacific Islander compared to non-Hispanic White infants.	Hispanic: aOR 1.27 (1.02–1.57) Asian or Pacific Islander: aOR 1.35 (1.01–1.80)	Birth year, gestational age, small for gestational age, multiple birth, Apgar, sex, required transfer, prenatal care, level of care, center, human milk at discharge

*aOR, adjusted odds ratio; aHR, adjusted hazards ratio; aRR, adjusted relative risk. Data in parentheses indicate 95% confidence intervals*.

For example, Guner et al. (16) performed a state-based analysis of NEC outcomes in California and used multivariate analysis to demonstrate that Hispanic ethnicity was independently associated with increased odds of death from NEC with adjusted odds ratio (aOR) of 1.50 and 95% confidence interval (CI) of 1.09–2.07. Two more recent studies of infants from California also showed similar findings of higher odds of NEC among Hispanics compared to non-Hispanic White infants (7, 17). Another study by Llanos et al. (14) analyzed NEC epidemiology from Upstate New York and found that non-Hispanic Black neonates had significantly higher risk of NEC compared to non-Hispanic White neonates even after adjusting for birth weight with relative risk (RR) of 1.70 (95% CI 1.06–2.61). A more recent study from New York that used a novel “fetus-at-risk” approach found that non-Hispanic Black infants had 4.4 times higher rate of NEC (95% CI 2.98–6.51) compared to non-Hispanic White infants (19).

Studies using US national databases demonstrated racial differences in NEC as well. An early study by Holman et al. (13) used data from the National Center for Health Statistics to study the epidemiology of NEC infant mortality in the United States and identified that non-Hispanic Black race was associated with higher mortality rate from NEC compared to non-Hispanic White race, even after controlling for birth weight, gestational age, and other characteristics (RR 1.5, 95% CI 1.3–1.8). Similar findings of increased incidence of NEC in non-Hispanic Black compared to non-Hispanic White infants were found in studies using the Kids' Inpatient Database (15) and the National Institute of Child Health and Human Development Neonatal Research Network (12). In a more recent study using the Pediatrix Medical Group database, Jammeh et al. (18) demonstrated that non-Hispanic Black and Hispanic infants are (1) significantly more likely to be diagnosed with NEC, and (2) have higher odds of death from NEC, compared to non-Hispanic White infants.

## Possible Explanations for Racial Disparities in NEC

### Biological Risk Factors That Mediate Association Between Race and NEC

**Figure 1:**
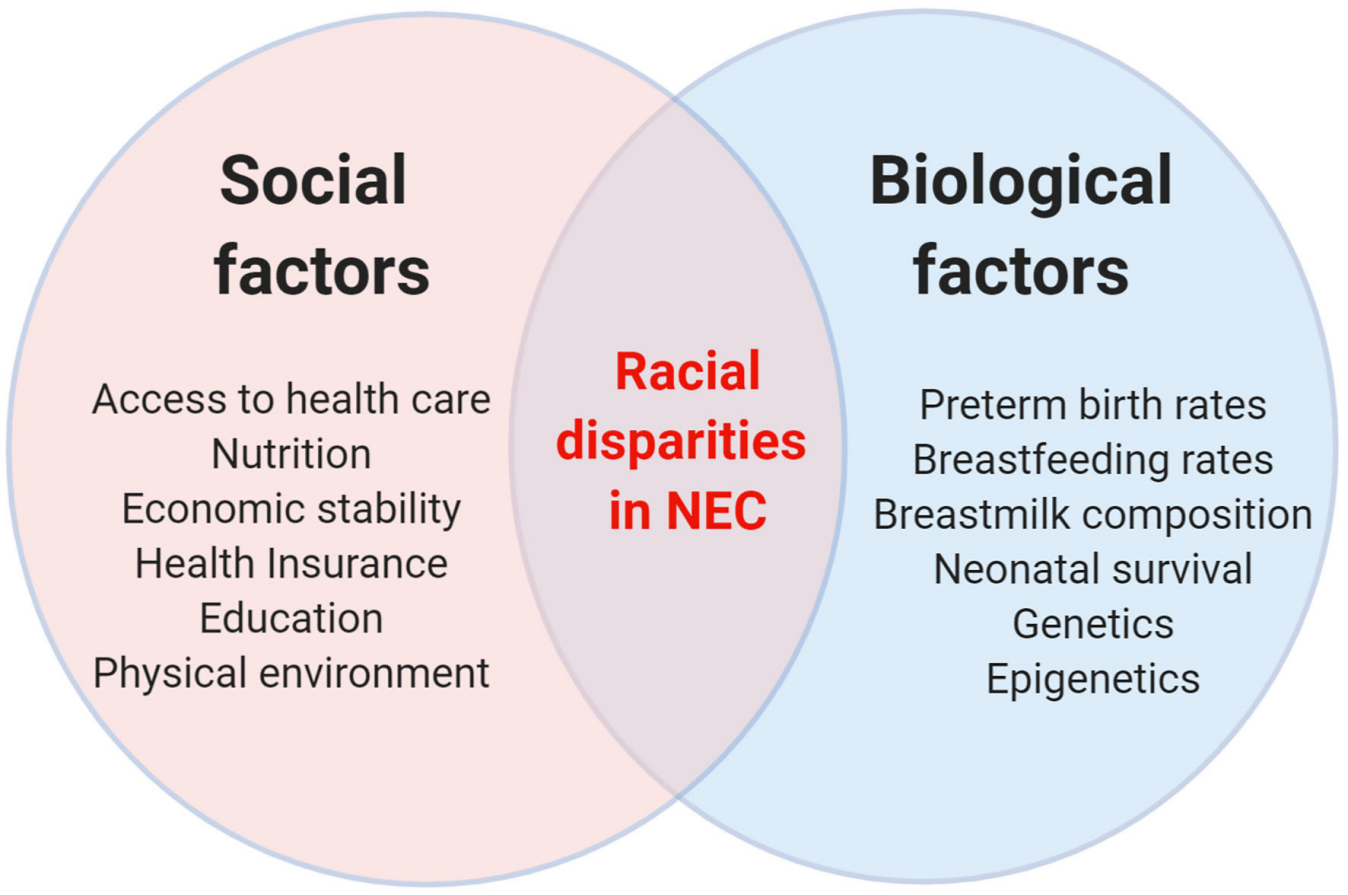
Schematic diagram showing the interplay between social and biological factors in driving racial disparities in NEC.

Racial disparities in NEC can be explained by its strong association with other biological risk factors known to impact NEC (Figure 1). For example, prematurity is identified as a clear and consistent risk factor for NEC. In a 2-year national surveillance study in England, NEC incidence was 11% among infants at 24 weeks gestation and decreased to 0.5% at 31 weeks gestation (20). Another important biologic risk factor for NEC is low birth weight. In one study from the Netherlands, small for gestational age infants have more than 2-fold increased risk for NEC compared to appropriate for gestational age neonates (21). As rates of pre-term birth and low birth weight infants are higher among non-Hispanic Black and Hispanic infants, it has been hypothesized that these factors, rather than race itself, mediate the higher rates of NEC observed in this racial population (22, 23). However, several studies that accounted for differences in birth weight and/or gestational age found that increased rates of NEC in non-Hispanic Black and Hispanic infants remained significant even after adjusting for these confounding factors (7, 13, 14, 16–18). Breastmilk feeding practices, a major protective factor against NEC, can also vary among different race groups. Population studies have observed that breastfeeding rates are lower among non-Hispanic Black infants compared to non-Hispanic White infants, which may help to explain the higher NEC rates among non-Hispanic Blacks (24, 25). The protective effect of breastmilk is highlighted in the recent study by Goldstein et al. (7) where higher odds of NEC in non-Hispanic Black infants compared to non-Hispanic White infants were no longer statistically significant when human milk use was accounted for. In their mediation analysis, human milk use accounted for 44% of the total risk of NEC in non-Hispanic Black vs. non-Hispanic White infants. Still, breastmilk use alone cannot account for the higher rates of NEC among Hispanics, who typically have higher breastfeeding rates than non-Hispanic Whites (26). A recent study by Gay et al. (27) also demonstrated differences in the actual composition of human milk metabolites among mothers from different countries. Whether such differences contribute to the differences in NEC rates among different racial or geographic populations remain unknown, but it is possible that the differences in breastmilk metabolites influence the infant's gut microbiota and mediate risk for NEC. Another plausible explanation is that because NEC and death are competing outcomes, the higher risk of neonatal mortality observed among non-Hispanic White pre-term infants can explain racial differences in NEC (28). In this hypothesis, non-Hispanic Black infants have higher NEC rates because of a greater number of survivors who remain at risk for NEC (13, 17, 18).

### Non-biological Mediators of Racial Disparities in NEC Outcomes

Race is also a strong proxy for social, cultural, educational, economic, and environmental experiences – all of which can influence disease risk (Figure 1). Thus, the observed racial differences in NEC may be explained by differences in non-biologic risk factors associated with race. For example, it is well-documented that Hispanics and non-Hispanic Blacks often have poor access to health care, including prenatal care (29, 30). Such racial disparities in prenatal care are important as several maternal factors have been associated with NEC, including premature rupture of membranes (31), chorioamnionitis (32), intrauterine growth restriction (33), placental abruption (34), and inadequate antenatal steroid administration for possible pre-term delivery (35). In another study, Kogan et al. (36) analyzed racial disparities in prenatal care and found that non-Hispanic Black women were more likely to report not receiving advice about important topics such as breastfeeding – a well-known protective mediator in NEC (37). Several studies have also identified inequality in the care of very low birth weight infants by race, with non-Hispanic Black and Hispanic infants receiving care at lower-quality hospitals compared to non-Hispanic White infants (38–41). While the exact reasons for variation in NEC incidence and outcomes across hospitals are unclear, clinical practice variables identified in the literature as potential protective mediators of NEC risk include use of standardized feeding protocols (42), donor breast milk (43), antibiotic stewardship (44), and probiotics (45). Other related variables that can contribute to higher NEC rates include the over-representation of adverse experiences among non-Hispanic Black infants such as poverty, malnutrition, stress, pollutants, and poor education. In addition to their impact on higher pre-term birth rates (46–48), these adverse experiences may also mediate racial differences in NEC outcomes through epigenetics and the microbiome (49, 50).

### Genetic Basis of Race-Dependent Differences in NEC Vulnerability

Another plausible explanation for the observed racial differences in NEC outcomes is that race imputes an innate biologic predisposition to NEC (Figure 1). This hypothesis is based on the assumption that different racial groups have unique patterns of genetic variations that can increase or decrease inherent risk for certain diseases (51). Genome wide sequencing studies evince that non-Hispanic Black populations have more heterozygote genotypes as well as more non-synonymous variants per individual compared to other racial populations (52, 53). Interestingly, selection pressures arising from infectious disease exposure has resulted in the non-Hispanic Black race having a stronger inflammatory response to pathogens (54). In adults, genetic programming of the immune response contributes up to a 3-fold difference in susceptibility to tuberculosis, septicemia and inflammatory diseases between non-Hispanic Black and non-Hispanic White populations (55, 56). Remarkably, NEC is also postulated to arise from an exaggerated inflammatory response to gut microbiota, and therefore racial differences in NEC vulnerability may be genetically programmed (4, 57).

Genetic studies in NEC have often been limited by small sample sizes and homogenous study populations with little racial diversity, limiting meaningful subgroup analysis by racial groups. One study by Franklin et al. (58) evaluated the association of several pro-inflammatory cytokine gene polymorphisms with NEC in a racially diverse population of pre-term infants from a single center in Washington, DC, and found that non-Hispanic White neonates with the C allele of IL-6 (rs1800795) were over 6 times more likely to have NEC than those with no C allele (*p* = 0.013; OR = 6.61, 95% CI 1.48–29.39). Another study by Sampath et al. (59) investigated autophagy genes in a multi-center cohort (*n* = 1,015) of pre-term infants with and without NEC and identified that the ATG16L1 (rs2241880, Thr300Ala) variant was associated with NEC. Risk of NEC was highest among infants with the AA genotype and decreased proportionately with the addition of the G allele (AA vs. GG: OR 2.5, 95% CI 1.2–5.6). In evaluating genetic differences along racial groups, the authors found that the proportion of infants with the protective GG allele was lower in non-Hispanic Black infants compared to non-Hispanic White infants (13 vs. 25%). Similarly, in another small study, NEC rates were higher in non-Hispanic Black pre-term infants, and correlated with higher prevalence of the NEC-associated NFKB1 (g.-24519delATTG) variant in that race (60).

These studies suggest that genetic variants which program increased inflammation are enriched in certain racial groups and may explain racial differences in NEC. Yet completion of the Human Genome Project has highlighted how humans are more similar than different from each other, and an ongoing debate remains about the importance of racial differences in genetic effects for disease (61–63). Comprehensive studies incorporating genetic, functional and clinical/demographic variables are required to characterize race-specific susceptible and resilience genetic factors.

## Discussion

While socioeconomic differences between races account for a substantial portion of racial disparity in health outcomes (64), adjusting for socioeconomic differences does not completely eliminate racial disparities in NEC. For example, Guner et al. (16) evaluated the contribution of household income and insurance status to differences in mortality from NEC in California and concluded that these socioeconomic factors could not explain the disparities in NEC mortality between Hispanic and non-Hispanic infants. Similarly, Janevic et al. (19) found that the increased risk among non-Hispanic Black infants for NEC remained despite including maternal educational level, insurance level, and other potential socioeconomic factors in their analysis of pre-term infants from New York. An important limitation of these studies that adjust for socioeconomic differences is the potential for unmeasured confounding by important variables that were not known or recorded in the original study database (65). Socioeconomic status is also difficult to measure, and misclassification or measurement errors in socioeconomic status are not uncommon (66, 67). Despite these limitations, these studies suggest that while social factors of race are certainly key contributors, these factors alone do not account for all the racial differences in NEC.

The contribution of genetic sequence differences between racial groups on risk of diseases such as NEC is also poorly understood (68). Emerging genetic studies of susceptibility to infection suggest that genetic variations can contribute to the host-inflammatory immune response (69–71). While some genetic studies in NEC have shown similar enrichment of pro-inflammatory genetic variants along racial groups, these studies should be considered exploratory and hypothesis-generating at best due to the limited sample size and lack of validation or functional studies (4, 72). The epigenetic effects of social and environmental factors on expression of key genes involved in NEC pathogenesis are also largely unexplored (73–75).

A better understanding of the contribution of race in NEC pathogenesis is needed to improve the racial disparities in NEC outcomes. Race is a complex trait that encompasses several social determinants and may also have important genetic underpinnings that contribute to NEC. In the search for a better understanding of how race impacts NEC, it is important to remember its multifaceted nature and avoid narrowly focusing on a single aspect while ignoring the rest.

## Conclusion

There is consistent epidemiological evidence for racial differences in NEC incidence and severity. While genetic variations across racial groups may result in biological effects that impact NEC pathogenesis, current genetic studies remain inadequate to prove or disprove this hypothesis. Race is a complex trait encompassing social, cultural, behavioral, and environmental dimensions that impact health and disease (76). In exploring racial differences in NEC, it is important to fully evaluate how both biological and non-biological aspects of race influence NEC pathogenesis independently as well as how they interact with each other. Alongside these research efforts, public health and quality improvement initiatives should also continue to actively reduce racial disparities in neonatal and perinatal care to improve outcomes.

## Author Contributions

AC and MK conceptualized the study. AC wrote the first draft of the manuscript. VS critically reviewed the manuscript and wrote the genetics section. All authors contributed to manuscript revision, read, and approved the submitted version.

## Conflict of Interest

The authors declare that the research was conducted in the absence of any commercial or financial relationships that could be construed as a potential conflict of interest.
